# Effects of Berberine on NLRP3 and IL-1*β* Expressions in Monocytic THP-1 Cells with Monosodium Urate Crystals-Induced Inflammation

**DOI:** 10.1155/2016/2503703

**Published:** 2016-09-05

**Authors:** Ya-Fei Liu, Cai-Yu-Zhu Wen, Zhe Chen, Yu Wang, Ying Huang, Sheng-Hao Tu

**Affiliations:** ^1^Institute of Integrated Traditional Chinese and Western Medicine, Tongji Hospital, Tongji Medical College, Huazhong University of Science and Technology, 1095 Jiefang Avenue, Wuhan, Hubei 430030, China; ^2^Department of Nephrology, The First Affiliated Hospital of Zhengzhou University, 1 Jianshe East Road, Zhengzhou, Henan 450052, China; ^3^Hubei University of Chinese Medicine, 1 Huangjiahu West Road, Wuhan, Hubei 430065, China

## Abstract

*Background.* Urate crystals-induced inflammation is a critical factor during the initiation of gouty arthritis. Berberine is well known for its anti-inflammatory activity. However, the underlying effects of berberine on monosodium urate crystals-induced inflammation remain obscure.* Objectives.* This study is set to explore the protective effect and mechanism of berberine on monosodium urate crystals-induced inflammation in human monocytic THP-1 cells.* Methods.* The mRNA levels of NLRP3 and IL-1*β* were measured by Real-Time PCR, and the protein levels of NLRP3 and IL-1*β* were determined by ELISA, Western blot, and immunofluorescence.* Results.* The NLRP3 and IL-1*β* expressions were significantly increased in model group compared to that in normal group (*P* < 0.05). Meanwhile, there was significant reduction in the expressions of NLRP3 and IL-1*β* mRNA in groups 6.25 *μ*M berberine and 25 *μ*M berberine when compared with model group (*P* < 0.05).* Conclusions. *Therefore, berberine alleviates monosodium urate crystals-induced inflammation by downregulating NLRP3 and IL-1*β* expressions. The regulatory effects of berberine may be related to the inactivation of NLRP3 inflammasome.

## 1. Introduction

Gout is characterized by hyperuricemia and triggered by the deposition of monosodium urate (MSU) crystals. The distribution of gout is imbalanced across the world, with prevalence being highest in Pacific countries. Developed countries incline to have a higher load of gout than developing countries and seem to have growing prevalence and incidence of gout [1]. The prevalence of both gout and hyperuricemia remains substantial, which is likely associated with increasing frequencies of adiposity and hypertension [2].

Gouty arthritis has self-resolution, with resolution within several days or 1-2 weeks. However, the pain is commonly unbearable. Urate-lowering therapies play a minor role in painful episodes. The IL-1*β* plays a key role in the acute gouty inflammation triggered by MSU crystals [3, 4]. Recent study showed that MSU crystals involved the activation of NALP3 inflammasome, bringing about the production of IL-1*β* [5]. Meanwhile, IL-1 inhibitors (rilonacept or anakinra) have been applied in patients with acute gouty arthritis [6, 7]. However, the relatively high medical care costs of IL-1 inhibitors restrict the application of these drugs in the developing countries.

Plant-based medicines are widely applied in treating gout and its complications in a number of hospitals in China. Among effective prescriptions, Simiao pill, which is derived from Ermiao powder and described in a famous traditional Chinese medicine monograph Chengfang Biandu in Qing Dynasty of China, has been wildly employed for treatment of gout and inflammatory arthritis [8]. Our previous study also demonstrated that modified Simiao decoction significantly decreased IL-1*β* release in THP-1 cells with MSU crystals-induced inflammation [9]. The ingredients of modified Simiao decoction are complicated, including alkaloids, flavones, and organic acids. Yin and colleagues have screened the active compounds of modified Simiao decoction with antigout effects [10, 11]. However, the specific ingredients and mechanisms of modified Simiao decoction functioning as antigout effects are still unknown.

*Cortex phellodendri*, referred to as “ruler drug,” plays a pivotal role in modified Simiao decoction. Berberine, the major component of* cortex phellodendri*, is able to lessen the expression of IL-1*β* in monocytes and macrophages [12, 13]. Based on the anti-inflammatory action of berberine, the study is to investigate the effects of berberine on NLRP3 and IL-1*β* in THP-1 cells with MSU crystals-induced inflammation on the basis of our previous research and to provide evidence for its application in gouty arthritis.

## 2. Materials and Methods

### 2.1. Reagents

Human IL-1*β* enzyme-linked immunosorbent assay (ELISA) kits were purchased from Beijing NeoBioscience Technology Co. Ltd., China. The RNAiso Plus (Code: D9108B), PrimeScript RT reagent Kit Perfect Real Time (Code: DRR036S), and SYBR Premix Ex Taq (Code: DRR420A) were purchased from TaKaRa Biotechnology Co. Ltd., Dalian, China. Fetal bovine serum was purchased from Sijiqing Biological Engineering Materials Co. Ltd. (Hangzhou, China). Berberine was obtained as a gift of Department of Integrated Traditional Chinese and Western Medicine (Tongji hospital, Huazhong University of Science and Technology). Tissue protein extraction kit, bicinchoninic acid (BCA) protein assay kit, and protease inhibitor cocktail were purchased from Wuhan Gugeshengwu Technology Co. Ltd. China. Rabbit polyclonal to IL-1*β* (Lot#: AP8531c), and Nlrp3 (Lot#: AP8564a) were purchased from Abgent Inc., USA. Rabbit anti-rat *β*-actin polyclonal antibody (Cat#: GB13001) was purchased from Wuhan Gugeshengwu Technology Co. Ltd., China. Fluorescent secondary antibody (Lot#: C30815-02) was obtained as a gift of Li-COR Inc., USA. FITC-labeled goat anti-rabbit secondary antibody and diamidino-phenylindole (DAPI) were purchased from Feiyi Technology Co. Ltd., Wuhan, and Boster Company, Wuhan, respectively. Uric acid sodium salt (product number: U2875) and phorbol 12-myristate 13-acetate (PMA) (product number: 79346) were ordered from Sigma-Aldrich Co., St. Louis, USA.

### 2.2. Main Devices

 The devices used include Mastercycler gradient PCR apparatus (Eppendorf Company, Germany); Nikon microimaging system (TE2000-U, Tokyo, Japan); Applied Biosystems StepOne Real-Time PCR System (Applied Biosystems, California, USA); microplate reader (BioTek Synergy2, Vermont, USA); inverted microscope (CKX-31, Olympus Corporation, Tokyo, Japan); CO_2_ incubator (New Brunswick Scientific Co. Inc., New Jersey, USA); Odyssey infrared imaging system (Li-COR Inc., USA); Nano Drop 2000 Spectrophotometer (Thermo Scientific Inc., Beijing, China).

### 2.3. Cell Culture

Monocytic THP-1 cells, human monocyte line, obtained as a gift of Department of Immunology (Tongji Medical College, Huazhong University of Science and Technology), were grown in RPMI-1640 medium supplemented with 10% heat-inactivated fetal bovine serum at 37°C and 5% CO_2_. THP-1 cells were plated at the density of 1.0–1.5 × 10^6^/mL in 6-well plates. THP-1 cells were stimulated for 3 h with 100 ng/mL PMA the day before stimulation. This treatment enhanced the phagocytic properties of the cells and prompted a constitutive production of pro-IL-1*β* [14]. THP-1 cells were stimulated with 100 *μ*g/mL MSU in the presence or absence of berberine. The prepared MSU solution should be kept at 4°C about one week before forming MSU crystals. THP-1 cells were randomized into normal group (N), model group (M), and berberine group (Ber) of different concentrations.

### 2.4. Real-Time PCR for IL-1*β* and NLRP3 mRNA Expressions

Total RNA was extracted from THP-1 cells with TRIzol reagent in accordance with the manufacturer's instructions. RNA concentration and purity were assessed by Nano Drop 2000 Spectrophotometer. One *μ*g of extracted total RNA was reverse transcribed with PrimeScript RT reagent Kit according to the manufacturer's instructions. The cDNA was stored at −20°C prior to PCR amplification. Real-Time PCR reactions were performed in 48-well optical PCR plates by employing an Applied Biosystems StepOne Real-Time PCR System in accordance with the manufacturer's instructions. 2^−ΔΔCT^ method was applied for analyzing the data. The primers were designed in accordance with published sequences (Table 1).

### 2.5. ELISA for IL-1*β* Protein Expression in Supernatants

The production of IL-1*β* was detected by quantitative sandwich enzyme immunoassay technique according to the manufacturer's standard protocols. The sensitivity of the ELISA kits of IL-1*β* was 4 pg/mL. None of the samples examined had a cytokine level >1000 pg/mL. The interassay and intra-assay coefficients of variation of the ELISA kits for IL-1*β* were less than 10%.

### 2.6. Western Blot for NLRP3 and IL-1*β* Protein Measurement

Total protein was extracted from THP-1 cells which were lysed in mammal tissue protein extraction reagent supplemented with protease inhibitor cocktail and then centrifuged at 12000 ×g for 10 min at 4°C. The BCA protein assay kit was applied to quantify the protein concentration of the supernatants. The total protein (*μ*g) was mixed with sample buffer, boiled for 10 min, and ran on a 12% or 10% SDS-PAGE gel. Separated proteins on the gel were transferred to polyvinylidene fluoride membranes. The membranes were blocked with 5% non-fat-dry milk for 1 h at room temperature and incubated overnight at 4°C with antibodies to *β*-actin, NLRP3, and IL-1*β* diluted 1 : 500, 1 : 150, and 1 : 100, respectively. The membranes were incubated in a lucifugous state with the fluorescent secondary antibody to rabbit diluted 1 : 5000 at room temperature for 1 h. Then, the membranes were detected by near infrared double-color laser imaging system. The pictures were analyzed with Odyssey software to calculate the gray scale ratio of *β*-actin.

### 2.7. Immunofluorescence for NLRP3

THP-1 cells were fixed on the coverslips. The samples were fixed with 4% paraformaldehyde in phosphate buffer saline (PBS) for 15 min at room temperature. The cells were washed three times with ice cold PBS. The samples were incubated for 15 min with PBS containing 0.1% Triton X-100. Cells were washed in PBS three times for 5 min. Cells were incubated with 10% goat serum for 20 min at room temperature to block unspecific binding of the antibodies. Cells were incubated in the diluted NLRP3 antibody (1 : 100) in a humidified chamber overnight at 4°C. The solution was decanted and the cells were washed three times in PBS, 5 min each wash. Cells were incubated with the diluted FITC-labeled secondary antibody (1 : 50) for 45 min at room temperature in the dark. The secondary antibody solution was decanted and the cells were washed three times with PBS in the dark, 5 min each wash. Cells were incubated with DAPI (DNA stain) for 3 min at room temperature in the dark. Coverslips were mounted with a drop of neutral balsam.

### 2.8. Statistical Analysis

All data with a normal distribution were presented as mean ± standard deviation (SD) and analyzed with aid of SPSS17.0 Statistical Software. Statistical significance was determined by one-way analysis of variance (ANOVA). For data with equal variances assumed, ANOVA followed by LSD test was applied. For data with equal variances not assumed, ANOVA followed by Dunnett's T3 test was used. A probability of less than 0.05 was considered to be statistically significant.

## 3. Results

### 3.1. Outcome of ELISA

**Figure 1:**
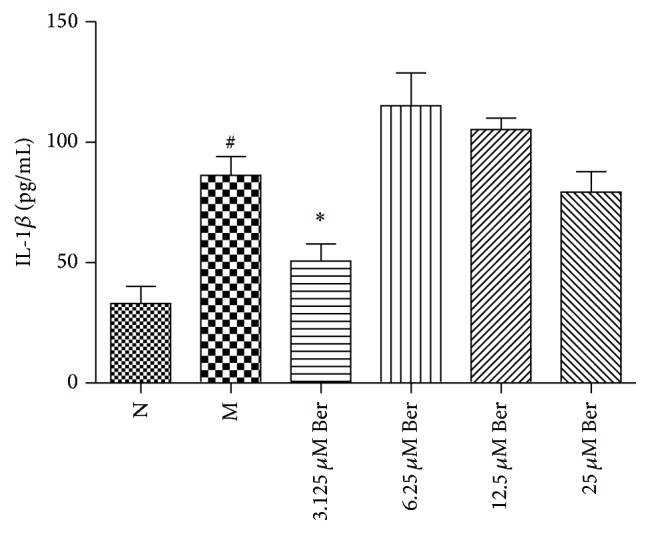
Expression of IL-1*β* in THP-1 cells. Values are mean ± SD. N: normal group; M: model group; 3.125 *μ*M Ber, 6.25 *μ*M Ber, 12.5 *μ*M Ber, and 25 *μ*M Ber: berberine group (Ber) of different concentrations. ^#^ *P* < 0.05 compared with group N; ^*∗*^ *P* < 0.05 compared with group M.

Compared with group N, the IL-1*β* level was significantly elevated in group M (*P* < 0.05). The IL-1*β* expression was significantly lowered in group 3.125 *μ*M Ber compared to that in group M (*P* < 0.05) (Figure 1). However, other concentrations of Ber did not lower the expression of IL-1*β* (*P* > 0.05).

### 3.2. Expression of IL-1*β* and NLRP3 mRNA in Lysates of THP-1 Cells

**Figure 2:**
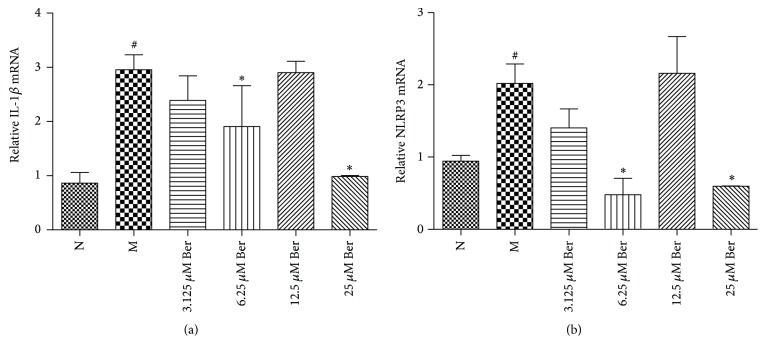
(a) Expression of IL-1*β* mRNA in THP-1 cells. (b) Expression of NLRP3 mRNA in THP-1 cells. Values are mean ± SD. N: normal group; M: model group; 3.125 *μ*M Ber, 6.25 *μ*M Ber, 12.5 *μ*M Ber, and 25 *μ*M Ber: berberine group (Ber) of different concentrations. NLRP3: domain present in neuronal apoptosis inhibitor protein major histocompatibility complex class II transactivator, leucine rich repeat, and pyrin domains-containing protein 3. ^#^ *P* < 0.05 compared with group N; ^*∗*^ *P* < 0.05 compared with group M.

As shown in Figure 2(a), IL-1*β* mRNA expression was significantly higher in group M compared to that in group N (*P* < 0.05). Compared with group M, there was a significant reduction in the expression of IL-1*β* in groups 6.25 *μ*M Ber and 25 *μ*M Ber. However, there was no significant reduction in the expression of IL-1*β* mRNA in groups 3.125 *μ*M Ber and 12.5 *μ*M Ber (*P* > 0.05).

As shown in Figure 2(b), NLRP3 mRNA expression was significantly higher in group M compared to that in group N (*P* < 0.05). Meanwhile, there was significant reduction in the expression of NLRP3 mRNA in groups 6.25 *μ*M Ber and 25 *μ*M Ber when compared with group M (*P* < 0.05). However, there was no significant reduction in the expression of NLRP3 mRNA in groups 3.125 *μ*M Ber and 12.5 *μ*M Ber (*P* > 0.05).

### 3.3. Outcome of Western Blot

**Figure 3:**
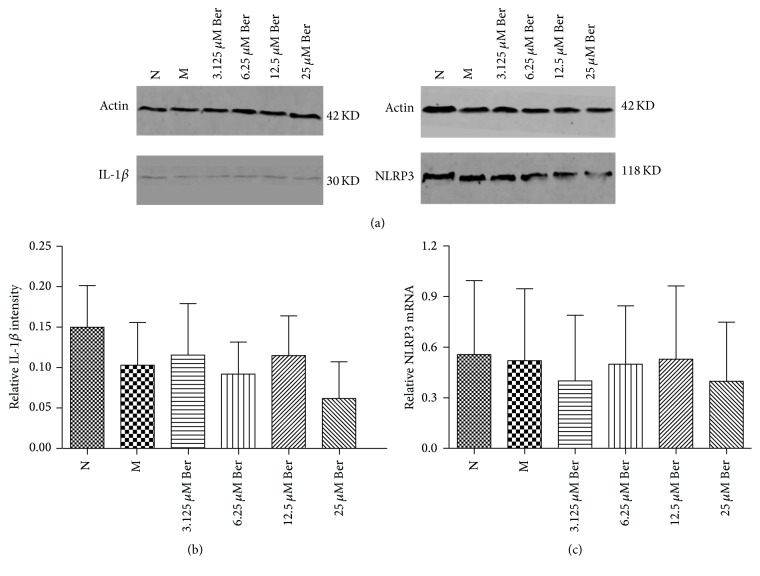
Outcome of Western blot (*n* = 4). (a) The representative bands of Western blot, (b) optical density of IL-1*β*, and (c) optical density of NLRP3. Values are mean ± SD. N: normal group; M: model group; 3.125 *μ*M Ber, 6.25 *μ*M Ber, 12.5 *μ*M Ber, and 25 *μ*M Ber: berberine group (Ber) of different concentrations. NLRP3: domain present in neuronal apoptosis inhibitor protein major histocompatibility complex class II transactivator, leucine rich repeat, and pyrin domains-containing protein 3.

Compared with group N, the IL-1*β* expression was decreased in group M; however, there was no significant difference between the two groups (*P* > 0.05). Compared with group M, the IL-1*β* expression was increased in groups 3.125 *μ*M Ber and 12.5 *μ*M Ber; however, there was no significant difference between the three groups (*P* > 0.05) (Figures 3(a) and 3(b)).

Compared with group N, the NLRP3 expression was nonsignificant alteration (*P* > 0.05). Compared with group M, the NLRP3 expression was blunted in groups 3.125 *μ*M Ber and 25 *μ*M Ber; however, there was no significant difference between the three groups (*P* > 0.05) (Figures 3(a) and 3(c)).

### 3.4. Immunofluorescence for NLRP3

**Figure 4:**
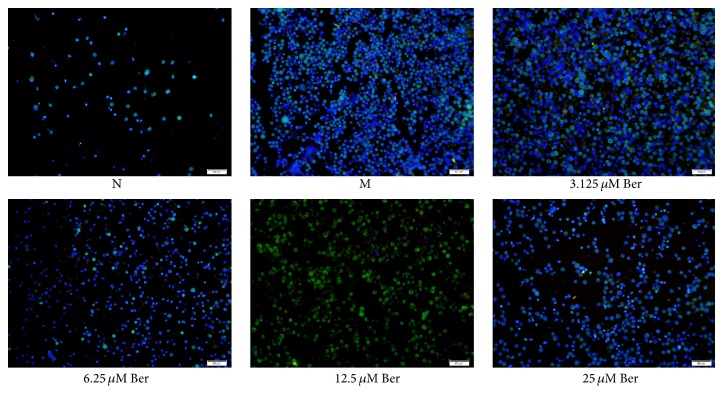
Immunofluorescence for NLRP3. N: normal group; M: model group; 3.125 *μ*M Ber, 6.25 *μ*M Ber, 12.5 *μ*M Ber, and 25 *μ*M Ber: berberine group (Ber) of different concentrations. NLRP3: domain present in neuronal apoptosis inhibitor protein major histocompatibility complex class II transactivator, leucine rich repeat, and pyrin domains-containing protein 3. The protein expression of NLRP3 was mainly located in the cytoplasm (green). The nuclei were stained with blue by DAPI.

NLRP3 was localized in the cytoplasm of THP-1 cells, stained with green. The nuclei were stained with blue by DAPI. The expression of NLRP3 in THP-1 cells was increased in group M compared to that in group N. Compared with group M, there was an decrease in the expression of NLRP3 in groups 6.25 *μ*M Ber and 25 *μ*M Ber (Figure 4).

## 4. Discussion

Traditional Chinese medicine is based on cumulative empirical experience of previous practitioners. A systematic review validated that Chinese herbal decoctions had similar clinical efficacy for the treatment of gout, but the Chinese herbal decoctions were superior to Western medicine in regard to attenuating adverse drug reactions [15]. Previous evidences from clinical practice and experimental studies confirmed that modified Simiao decoction had the potential to treat hyperuricemia and gouty arthritis [14, 16, 17]. Ultrafiltration liquid chromatography combined with high-speed countercurrent chromatography screened and isolated *α*-glucosidase and xanthine oxidase inhibitors from* Cortex phellodendri* for the prevention and treatment of gout [18]. However, previous studies focused on hypouricemic effects of modified Simiao decoction. To our knowledge, gouty arthritis could attack patients with a serum uric acid concentration in the normal range.

Therefore, it is necessary to inhibit the cytokines release in acute phase of gouty arthritis. Ankle joint urate arthritis provided a useful tool for the evaluation of anti-inflammatory and antigout agents [19, 20]. However, MSU crystals-induced inflammation model in monocytes was rarely reported [5]. In our previous study, the MSU crystals-induced inflammation model in THP-1 cells was successfully established by the stimulation of PMA and MSU [9].

Innate immunity provides the first line of defense against pathogen-associated molecular patterns (PAMPs) and damage associated molecular patterns (DAMPs) via primitive responses that are nonspecific and broad in spectrum. MSU crystals may act as PAMP or DAMP and are recognized by the pattern recognition receptors of the innate immune system. Unlike adaptive immunity, innate immune responses do not directly generate immunologic memory or lasting protective immunity, in line with the nature of recurrent acute gouty attack [21]. NLRP3 inflammasome plays an important role in MSU crystals-induced innate immune responses.

**Figure 5:**
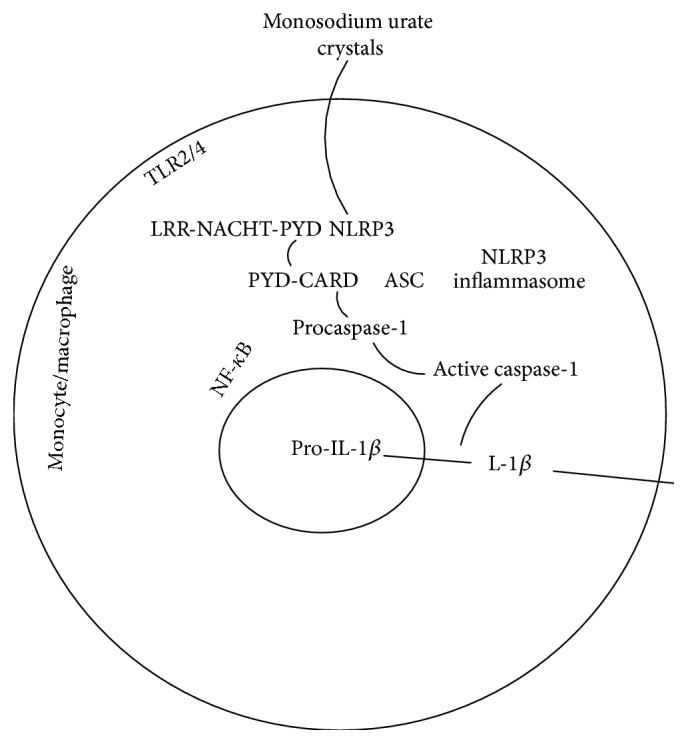
NLRP3 and IL-1*β* signalling in gout. Intracellular monosodium urate crystals are recognized by NLRP3 inflammasome, leading to oligomerization of NLRP3 and cleavage of procaspase-1 to caspase-1. Further, caspase-1 cleaves inactive pro-IL-1*β* to produce active IL-1*β*. NLRP3, domain present in neuronal apoptosis inhibitor protein major histocompatibility complex class II transactivator, leucine rich repeat, and pyrin domains-containing protein 3; TLR, Toll-like receptor; ASC, apoptosis-associated speck-like protein containing a caspase-recruitment domain [CARD]; NLR, NOD-like receptor; PYD, pyrin domain; LRR, leucine rich repeat; NACHT, domain present in neuronal apoptosis inhibitor protein major histocompatibility complex class II transactivator; NF-*κ*B, nuclear factor-*κ*B.

NLRP3 inflammasome is composed of leucine rich repeat- (LRR-) containing Nlrp3, an adaptor protein such as apoptosis-associated speck-like protein containing a caspase activation and recruitment domain (ASC) and an effector caspase-1 which activates proinflammatory cytokines. Intracellular MSU crystals are recognized by NLRP3 inflammasome, giving rise to oligomerization of NLRP3 and cleavage of procaspase-1 to caspase-1. Further, caspase-1 cleaves inactive pro-IL-1*β* to produce active IL-1*β* [22] (Figure 5).

Our results were consistent with Liu et al. in terms of berberine in reducing IL-1*β* release with lipopolysaccharide-induced inflammation [12]. Unlike the dose-effect relationship of Liu et al., high dose of berberine was unable to diminish the IL-1*β* expression. The difference may result from berberine purity and diverse inflammation models. In accordance with the outcome of ELISA, there was a significant increase in the expression of IL-1*β* mRNA in group M when compared with group N. However, different concentrations of berberine had different effects on transcription and translation level of IL-1*β* mRNA. Posttranscriptional processing and degradation and posttranslational processing and modification may contribute to the discrepancies.

With regard to the expression of NLRP3 mRNA, our findings were consistent with Dang et al. who found that mRNA expression of NLRP3-2, NLRP3-3, and NLRP3-4 increased significantly in the patients with acute gouty arthritis and nonacute gouty arthritis compared with healthy controls [23]. However, Yang et al. showed that the expression of NLRP3 mRNA was significantly lower in patients with acute gouty arthritis compared with healthy controls [24]. The differences may result from different models* in vivo *and* in vitro*.

IL-1*β* is a secretive protein, and hence Western blot via cell lysates may determine the pro-IL-1*β*. Our results showed that different concentrations of berberine had no effects on IL-1*β*, which was different from the result of ELISA. The differences may result from determination of inactive or active IL-1*β*.

The outcomes of Western blot showed that different concentrations of berberine had no effects on NLRP3 expression, which was different from the result of Real-Time PCR. Semiquantitative method of Western blot, processing, and modification of transcription and translation may result in the divergences. NLRP3 was localized in the cytoplasm of THP-1 cells from immunofluorescence. The result of immunofluorescence was consistent with NLRP3 mRNA in terms of lowering the expression of NLRP3.

The different concentrations of berberine may regulate the expressions of mRNA and protein of NLRP3 and IL-1*β* through NLRP3 inflammasome and downstream signalling molecules, resulting in attenuating the MSU crystals-induced inflammation.

## 5. Conclusions

Berberine may influence the NLRP3 inflammasome which is involved in MSU crystals-induced innate immune responses, attenuate the expression of NLRP3, further inhibit downstream signalling molecular IL-1*β*, and eventually play a role in treatment of gouty arthritis. The effects and mechanisms of berberine on NLRP3 inflammasome related LRR, ASC, and caspase-1 need to be further validated.

## Figures and Tables

**Table 1:** Primer sequence.

Gene	Primer	Sequence
*β*-actin	Forward	5′-TGGCACCCAGCACAATGAA-3′
	Reverse	5′-CTAAGTCATAGTCCGCCTAGAAGCA-3′

IL-1*β*	Forward	5′-GCTGATGGCCCTAAACAGATGAA-3′
	Reverse	5′-TCCATGGCCACAACAACTGAC-3′

TNF-*α*	Forward	5′-TGCTTGTTCCTCAGCCTCTT-3′
	Reverse	5′-CAGAGGGCTGATTAGAGAGAGGT-3′

NLRP3	Forward	5′-GTCATCGGGTGGAGTCACTGTC-3′
	Reverse	5′-AAGTGAGGTGGCTGTTCACCAA-3′

NLRP3: domain present in neuronal apoptosis inhibitor protein major histocompatibility complex class II transactivator, leucine rich repeat, and pyrin domains-containing protein 3.
